# Supplementary material for: Assessment of the Effects of Chitosan, Chitooligosaccharides and Their Derivatives on Lemna minor
Source: Molecules. 2022 Sep 19;27(18):6123. doi: 10.3390/molecules27186123 (PMC9502776; doi:10.3390/molecules27186123)
Supplement: Supplementary file 1 [file molecules-27-06123-s001.zip › molecules-1895038-supplementary.pdf]

Supplementary materials

Table S1. Results of Dunn's posthoc test for D-glucosamine. Statistically significant data ($p < 0.05$) are written in bold

	5 mg/L	50 mg/L	500 mg/L
**5 mg/L**		**0.034**	**0.034**
**50 mg/L**			1
**500 mg/L**			

Table S2. Results of Tukey's posthoc test for N-acetyl-D-glucosamine (above diagonal) and for chitobiose dihydrochloride (below diagonal). Statistically significant data ($p < 0.05$) are written in bold

	5 mg/L	50 mg/L	500 mg/L
**5 mg/L**		0.596	0.706
**50 mg/L**	0.354		0.979
**500 mg/L**	0.081	**0.013**	

Table S3. Results of Dunn's posthoc test for N-carboxymethyl chitosan. Statistically significant data ($p < 0.05$) are written in bold

	50 mg/L	250 mg/L	500 mg/L	5000 mg/L
**50 mg/L**		0.364	0.427	**0.009**
**250 mg/L**			0.910	0.089
**500 mg/L**				0.070
**5000 mg/L**				

Table S4. Results of Dunn's posthoc test for Low MW chitosan (above diagonal) and of Tukey's posthoc test for Medium MW chitosan (below diagonal). Statistically significant data ($p < 0.05$) are written in bold

	50 mg/L	500 mg/L	5000 mg/L
**50 mg/L**		0.285	**0.033**
**500 mg/L**	**0.002**		0.285
**5000 mg/L**	**0.001**	**0.007**	

Table S5. Results of Tukey's posthoc test for High MW chitosan (above diagonal) and for chitosan with ≈ 50% DaD (below diagonal). Statistically significant data ($p < 0.05$) are written in bold

	50 mg/L	500 mg/L	5000 mg/L
**50 mg/L**		**0**	**0**
**500 mg/L**	**0.001**		**0**
**5000 mg/L**	**0.001**	0.386	

Table S6. Molecular weight (MW) and deacetylation degree (DaD) of chitooligosaccharide (CO), CO derivative and chitosan samples

Tested samples	Sample ID	MW (kDa)	DaD (%)
D-glucosamine hydrochloride	G	0,216	0
N-acetyl-D-glucosamine	NAG	0,221	100
chitobiose dihydrochloride	2G	0,413	0
N-carboxymethyl chitosan	CMChi	0,544	90
Low MW chitosan	ChiS	198	81
Medium MW chitosan	ChiM	307	84
High MW chitosan	ChiL	604	83
Chitosan with ≈ 50% DaD	Chi50	278	48

Table S7. Results of Pearson Linear r correlation test for EC_{50} values and molecular weight of various sample combinations. Statistically significant data ($p < 0.05$) are written in bold

Correlation of EC_{50} and MW of	*p* value	Correlation statistic
G, NAG, 2G, CMChi, ChiS, ChiM, ChiL, Chi50	0.996	-0.002
G, NAG, 2G	0.930	-0.110
G, NAG, 2G, CMChi	0.422	0.578
G, 2G, CMChi	0.241	0.929
ChiS, ChiM, ChiL, Chi50	0.327	0.673
CMChi, ChiS, ChiM, ChiL, Chi50	0.489	-0.413
G, 2G, ChiS, ChiM, ChiL	0.062	0.858
2G, ChiS, ChiM, ChiL	**0.011**	0.988
Chi50, ChiS, CMChi	**0.019**	-0.999

Table S8. Results of Pearson Linear r correlation test for EC_{50} values and deacetylation degree of various sample combinations. Statistically significant data ($p < 0.05$) are written in bold

Correlation of EC_{50} and MW of	*p* value	Correlation statistic
G, NAG, 2G, CMChi, ChiS, ChiM, ChiL, Chi50	0.536	0.258
G, NAG, 2G	0.247	0.926
G, NAG, 2G, CMChi	0.596	0.404
ChiS, ChiM, ChiL, Chi50	0.100	-0.900
CMChi, ChiS, ChiM, ChiL, Chi50	0.141	-0.754
G, ChiS, ChiM, ChiL, NAG	0.176	0.713
2G, ChiS, NAG	0.088	0.990
ChiS, ChiM, ChiL, CMChi	0.051	-0.949
ChiS, ChiL, CMChi	**0.044**	-0.997

Table S9. Results of Pearson Linear r correlation test for Wiener index, molecular weight (MW), log P, log S, IGC_{50}, $LC_{50}FM$ and $LC_{50}DM$ for the analyzed chitooligosaccharides (unprotonated structures) and their derivatives. Correlation statistics are shown below the diagonal and *p* values are shone above the diagonal, with statistically significant data ($p < 0.05$) being written in bold

	**Wiener Index**	**MW**	**logP**	**logS**	**IGC_{50}**	**$LC_{50}FM$**	**$LC_{50}DM$**
**Wiener Index**		**1.20E-09**	**0.001**	**0.016**	**0.005**	**0.002**	0.213
**MW**	0.919		**1.30E-05**	**1.22E-05**	**0.003**	0.371	**0.031**
**logP**	-0.781	-0.844		**4.08E-07**	0.973	0.089	0.145
**logS**	0.735	0.845	-0.856		0.433	0.159	**0.043**
**IGC_{50}**	-0.481	-0.504	-0.006	-0.144		0.662	**2.89E-12**
**$LC_{50}FM$**	-0.526	-0.662	0.305	-0.684	0.646		0.094
**$LC_{50}DM$**	-0.677	-0.722	0.264	-0.359	0.946	0.697	

Table S10. Results of Pearson Linear r correlation test for Wiener index, molecular weight (MW), log P, log S, IGC_{50}, $LC_{50}FM$ and $LC_{50}DM$ for the analyzed chitooligosaccharides (protonated structures). Correlation statistics are shown below the diagonal and *p* values are shone above the diagonal, with statistically significant data ($p < 0.05$) being written in bold

	**Wiener Index**	**MW**	**logP**	**logS**	**IGC_{50}**	**$LC_{50}FM$**	**$LC_{50}DM$**
**Wiener Index**		**1.62E-10**	**0.002**	0.112	**0.006**	**0.002**	0.205
**MW**	0.917		**1.82E-05**	**8.95E-05**	**0.004**	0.287	**0.031**
**logP**	-0.771	-0.840		**1.01E-05**	0.909	0.084	0.155
**logS**	0.693	0.821	-0.847		0.560	0.153	0.069
**IGC_{50}**	-0.476	-0.497	-0.021	-0.107		0.066	**4.48E-12**
**$LC_{50}FM$**	-0.529	-0.669	0.310	-0.685	0.646		0.090
**$LC_{50}DM$**	-0.677	-0.722	0.257	-0.326	0.945	0.698	
